# Identification of a specific agonist of human TAS2R14 from *Radix Bupleuri* through virtual screening, functional evaluation and binding studies

**DOI:** 10.1038/s41598-017-11720-0

**Published:** 2017-09-22

**Authors:** Yuxin Zhang, Xing Wang, Xi Li, Sha Peng, Shifeng Wang, Christopher Z. Huang, Corine Z. Huang, Qiao Zhang, Dai Li, Jun Jiang, Qin Ouyang, Yanling Zhang, Shiyou Li, Yanjiang Qiao

**Affiliations:** 1 Key Laboratory of TCM-information Engineer of State Administration of TCM, School of Chinese Pharmacy, Beijing University of Chinese Medicine, No. 6, Central Ring South Road, Wangjing, Beijing, 100102 China; 2 Key Laboratory of Genomic and Precision Medicine, Beijing Institute of Genomics, Chinese Academy of Sciences, Beichen West Road, No. 1, Chaoyang District, Beijing, 100101 China; 3 Beijing Key Lab of Traditional Chinese Medicine Collateral Disease Theory Research, School of Traditional Chinese Medicine, Capital Medical University, Fengtai District, Beijing, 100069 China; 4 HD Biosciences, Co., Ltd. 590 Ruiqing Road, Zhangjiang Hi-Tech Park East Campus, Pudong New Area, Shanghai, 201201 China; 5 Chinese International School, 1 Hau Yuen Path, Braemar Hill, Hong Kong, SAR China; 6 School of Pharmacy, The Third Military Medical University, Gaotanyan Street, No. 30, Chongqing, 400038 China

## Abstract

Bitter taste receptors (TAS2Rs) have attracted a great deal of interest because of their recently described bronchodilator and anti-inflammatory properties. The aim of this study was to identify natural direct TAS2R14 agonists from *Radix Bupleuri* that can inhibit mast cell degranulation. A ligand-based virtual screening was conducted on a library of chemicals contained in compositions of *Radix Bupleuri*, and these analyses were followed by cell-based functional validation through a HEK293-TAS2R14-G16gust44 cell line and IgE-induced mast cell degranulation assays, respectively. Saikosaponin b (SSb) was confirmed for the first time to be a specific agonist of TAS2R14 and had an EC_50_ value of 4.9 μM. A molecular docking study showed that SSb could directly bind to a TAS2R14 model through H-bond interactions with Arg160, Ser170 and Glu259. Moreover, SSb showed the ability to inhibit IgE-induced mast cell degranulation, as measured with a β-hexosaminidase release model and real-time cell analysis (RTCA). In a cytotoxicity bioassay, SSb showed no significant cytotoxicity to HEK293 cells within 24 hours. This study demonstrated that SSb is a direct TAS2R14 agonist that inhibit IgE-induced mast cell degranulation. Although the target and *in vitro* bioactivity of SSb were revealed in this study, it still need *in vivo* study to further verify the anti-asthma activity of SSb.

## Introduction

Human perception of bitter taste is mediated by taste type 2 receptors (TAS2Rs), which are the expression products of the 25 human TAS2R genes^1^. TAS2Rs were first identified in taste buds and were subsequently discovered in extra-oral systems, where they acted with various physiological effects^2^. Among these extra-oral regions, the expression and potential functional roles of TAS2Rs in airways have attracted a great deal of interest^2–10^. Previous studies have reported that TAS2R14, the first receptor identified to exhibit a broad agonist spectrum, has the highest expression level in human bronchi among the 25 TAS2Rs^6^. TAS2R14 agonists, such as quinine, caffeine and diphenidol, have been reported to induce significant effects on airway smooth muscle relaxation^6,11,12^. Moreover, direct TAS2R14 agonists such as noscapine can inhibit IgE-dependent mast cell activation^12,13^, which was thought to be a promising approach to allergic asthma treatment. Thus, TAS2R14 has great potential as a therapeutic target against respiratory diseases. Consequently, determining direct TAS2R14 agonists could be valuable for treating respiratory diseases and exploring their biological mechanisms.

Natural products have been considered a valuable source for small-molecule drug discovery^14,15^. *Radix Bupleuri*, a common Chinese herbal medicine designated as “bitter taste” according to Chinese medicine theory, displays anti-asthmatic and antitussive effects in clinical use^16^. It has been reported that many classic *Radix Bupleuri*-based traditional Chinese formulae have good clinical effects in the treatment of acute and chronic bronchitis^17,18^. The main active ingredients of *Radix Bupleuri* include triterpenoid glycosides of saikosaponin, essential oils and polysaccharides^19^. Saikosaponins, such as the most prevalent examples, saikosaponin a (SSa), saikosaponin b (SSb), saikosaponin c (SSc) and saikosaponin d (SSd), are recognized as the principal bioactive components^20,21^. To explore which ingredients trigger the bitter taste and whether the bitter tastants are related to the clinical effect of *Radix Bupleuri*, we collected 155 compounds for this study. A TAS2R14 subtype was selected for study among the 25 TAS2Rs, with consideration for the highest expression level in human bronchi and the broadest agonist spectrum combined with the functional efficacy of the agonists. Thus, this study was devoted to identifying direct TAS2R14 agonists from *Radix Bupleuri* and evaluating their efficacy in inhibiting mast cell degranulation, which is an important mechanism in allergic asthma. Ligand-based virtual screening combined with a HEK293-TAS2R14-G16gust44 cell-based calcium functional assay was implemented to search for direct TAS2R14 agonists in the chemical database of *Radix Bupleuri*. The newfound agonist was then evaluated to determine the inhibition effects on IgE-induced mast cell degranulation. Molecular docking was carried out to characterize the interaction between the agonist and TAS2R14. This study provided an efficient strategy to identify TAS2R14 agonists from natural products, which could promote the development of safe and effective anti-asthmatic agents from natural resources.

## Results

### Virtual screening for direct TAS2R14 agonists

**Figure 1:**
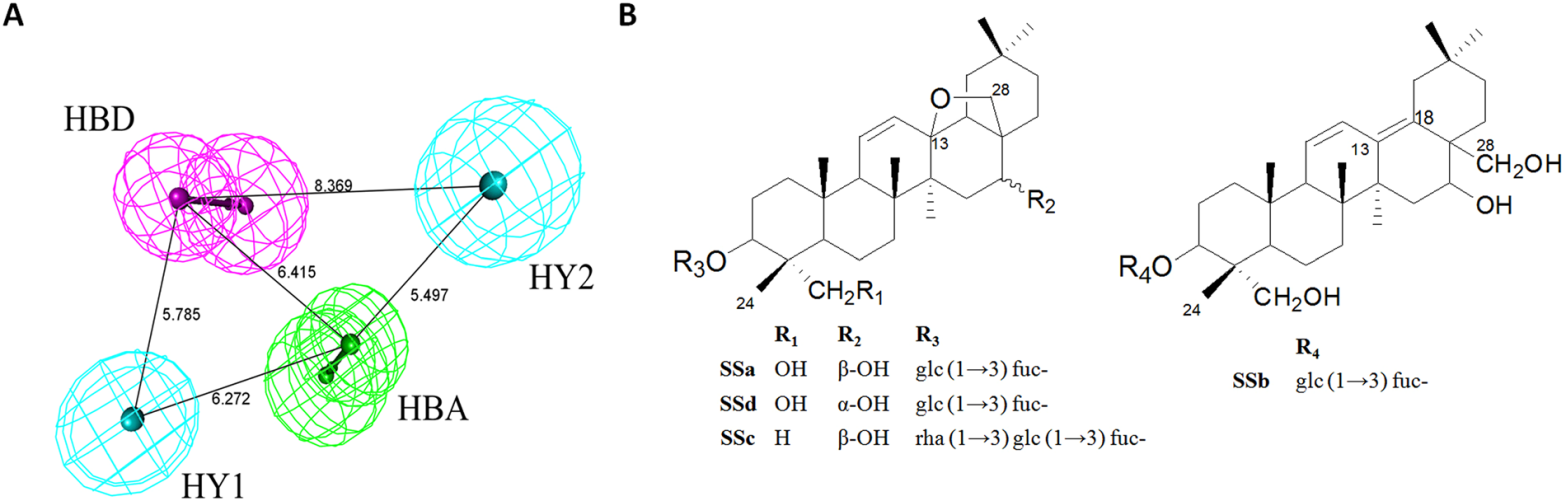
Pharmacophore models and structures of saikosaponins. (**A**) Pharmacophore Model_01 of TAS2R14 agonists. The numbers represent the distance between two pharmacophore features. The arrows represent the direction of hydrogen bond groups. Gray, white, red, blue and yellow spheres represent carbon, hydrogen, oxygen, nitrogen and sulfur atoms, respectively. HY: hydrophobic portions; HBD: hydrogen bond donor; HBA: H-bond acceptors. (**B**) Structures of saikosaponins (SSa, SSb, SSc and SSd). SSa, SSc and SSd are epoxy ether derivatives, whose 13, 28β-epoxy ether bond is key characteristic. SSb is a heterocyclic diene derivative whose hydroxymethyl group is at the C-28 position.

Ten pharmacophore models of TAS2R14 agonists were generated and evaluated using the built-in parameters (Table 1). Model_01 (Fig. 1A), which had the highest comprehensive appraisal index (CAI)^22,23^, was considered the best model with which to identify active compounds and exclude inactive compounds comprehensively. Model_01 contains two hydrophobic portions (HY), one hydrogen bond donor (HBD) and one hydrogen bond acceptors (HBA). Model_01 was used as a three-dimensional (3D) query to screen the self-built 3D Chemical Database of *Radix Bupleuri* (RBDB), producing a list of eight candidates (Table 2). Among the candidates, SSa, SSb, SSc and SSd belonged to the set of saikosaponins with fit values above 0.9. Saikosaponins, a subset of the pentacyclic triterpenoids, are made up of triterpenoid sapogenin and sugar units. SSa, SSc and SSd are epoxy ether derivatives, whose 13, 28β- epoxy ether bond is their key characteristic, while SSb is a heterocyclic diene derivative and holds hydroxymethyl group at the C-28 position (Fig. 1B).

**Table 1:** TAS2R14 Pharmacophore model calculation results.

Model	A^a^	D^b^	Ht^c^	Ha^d^	A%^e^	N^f^	CAI^g^
01	37	228	42	20	54.1	2.93	1.59
02	37	228	40	19	51.4	2.93	1.50
03	37	228	41	19	51.4	2.86	1.47
04	37	228	39	19	51.4	3.00	1.54
05	37	228	43	20	54.1	2.87	1.55
06	37	228	44	18	48.6	2.52	1.23
07	37	228	47	20	54.1	2.62	1.42
08	37	228	46	19	51.4	2.55	1.31
09	37	228	45	19	51.4	2.60	1.34
10	37	228	42	17	45.9	2.49	1.15

^a^A represents the number of known TAS2R14 agonists in the test database; ^b^D represents the total number of compounds in the test database; ^c^Ht is the total number of hits through pharmacophore-based virtual screening; ^d^Ha is the number of active hits through pharmacophore-based virtual screening; ^e^A% implies the ability for the pharmacophore model to identify TAS2R14 agonists from the test database; ^f^N implies the ability to distinguish TAS2R14 agonists from non-TAS2R14 agonists; ^g^CAI represents the comprehensive appraisal index.

**Table 2:** The hits through pharmacophore-based virtual screening.

ID	Molecular Formula	Names	^a^Fit Value	Matching model
1	C_48_H_78_O_17_	Saikosaponin^c^	0.997	
2	C_42_H_68_O_13_	Saikosaponin^b^	0.996	
3	C_42_H_68_O_13_	Saikosaponin^a^	0.993	
4	C_42_H_68_O_13_	Saikosaponin^d^	0.988	
5	C_21_H_20_O_12_	Isoquercitrin	0.750	
6	C_16_H_12_O_5_	Wogonin	0.718	
7	C_10_H_12_O_2_	Eugenol	0.500	
8	C_11_H_6_O_3_	Angelicin	0.494	

^a^Fit Value is a score to evaluate the fitness and dependence on the proximity of the features to pharmacophore centroids and the weights assigned to each feature. The closer this value is to 1, the better match it is.

### *In vitro* identification of TAS2R14 agonists

**Figure 2:**
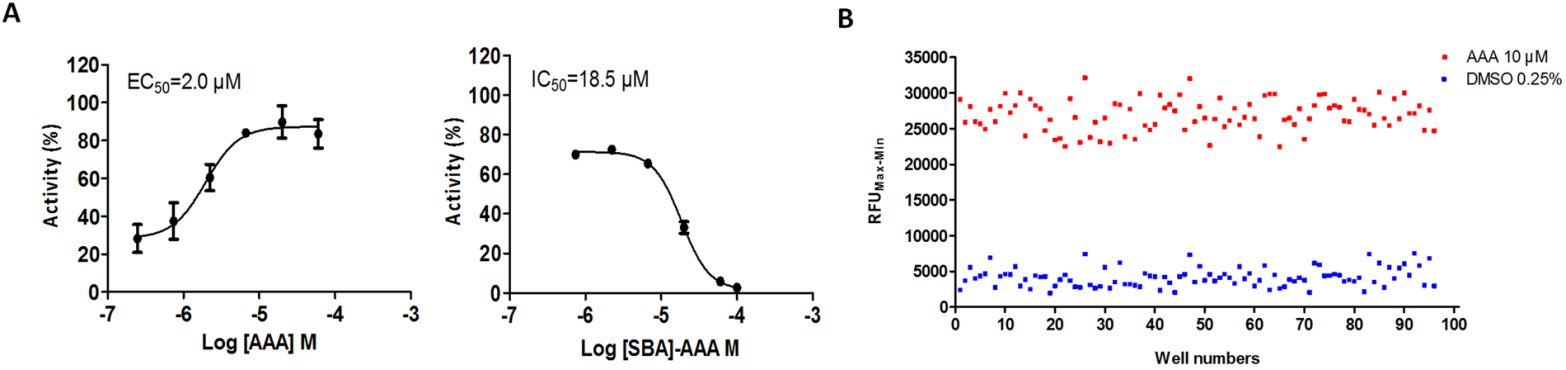
TAS2R14-G16gust44-HEK293 cell line establishment. (**A**) Dose-response curves of a TAS2R14 agonist (aristolochic acid A, AAA). The EC_50_ value of AAA was 2.0 μM. Dose-response curves of TAS2R14 antagonist (SBA). The IC_50_ value of SBA stimulated in the presence of 10.0 μM AAA was determined to be 18.5 μM. The maximum RFU value of TAS2R14 activation was set at 100%, and the minimum was set at 0%. All error bars indicate the SE of three replicates. The EC_50_ and IC_50_ values were determined in normalized RFU (relative fluorescence units) using GraphPad Prism 5 software. (**B**) In the Z′ factor evaluation experiments, AAA (10 μM) and 0.25% (v/v) DMSO were used as positive and negative controls, respectively. The Z′ factor value of the high-throughput screening assay was 0.54 (>0.50), which indicates good separation of the distributions. RFU_Max-Min_ means the difference in relative fluorescence units between the maximum and the minimum.

A recombinant HEK293-TAS2R14-G16gust44 cell-based calcium mobilization assay was established to verify direct TAS2R14 agonists based on virtual screening. The EC_50_ value of aristolochic acid A (AAA), a known TAS2R14 agonist, was determined to be 2.0 μM. The IC_50_ of sulfamoyl-benzoic acid (SBA), a known TAS2R14 antagonist^24^, in the presence of 10.0 μM AAA was determined to be 18.5 μM (Fig. 2A). Moreover, the Z′ factor of the TAS2R14-G16gust44 agonist screening assay was determined to be 0.54, which indicated good separation of the distributions (Fig. 2B)^25^. The results indicated that the established HEK293-TAS2R14-G16gust44 cell line could effectively evaluate the agonistic activity of TAS2R14.

**Figure 3:**
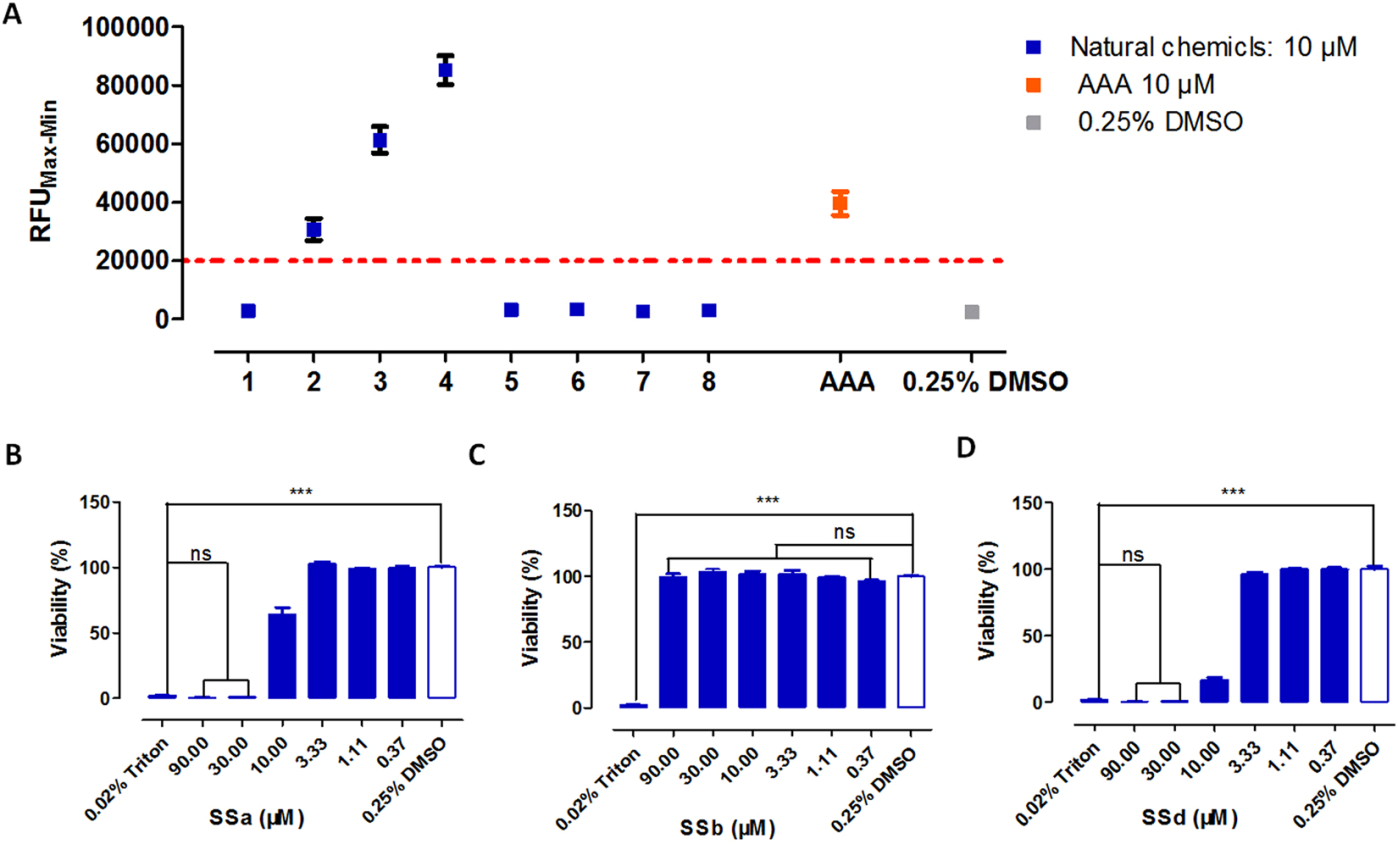
Calcium imaging and cytotoxicity evaluation of potential TAS2R14 agonists. (**A**) Calcium imaging of eight natural chemical hits (10 μM) from virtual screening was conducted in the HEK293-TAS2R14-G16gust44 cell line. All error bars indicate the SE of three replicates. HEK293-TAS2R14-G16gust44 cells were treated with different concentrations of SSa (**B**), SSb (**C**), SSd (**D**) and 0.25% DMSO in 5% CO_2_ at 37 °C for 24 hours. A concentration of 0.02% Triton was added 10 min before detection for the sensitivity evaluation experiment. Luminescence was read with Envision 2100 multilabel reader to detect viability following incubation with CellTiter-Glo reagent for 15 min. Compared with the control group, SSb showed no significant cytotoxicity to HEK293 cell within 24 hours. SSa and SSd showed significant toxicity at high concentrations. RFU_Max-Min_ means the difference in relative fluorescence units between the maximum and the minimum. All error bars indicate the SE of three replicates. ns means there was no significant difference between the control group and the AAA groups; ***means p < 0.05.

In the primary test, eight candidates were evaluated through the cell-based calcium mobilization assay at a concentration of 10 μM. Among the eight compounds, only three compounds, namely SSa, SSb and SSd, showed significantly greater signals than the control group (***p < 0.05) in the Ca^2+^ influx assay (Fig. 3A).

### Compound cytotoxicity evaluation

A quantitative luciferase-coupled adenosine triphosphate (ATP) assay was used to determine the cytotoxicity of the hits (SSa, SSb and SSd). Compared with the control group, SSb showed no cytotoxicity in HEK293 cell within 24 hours (Fig. 3C), while SSa and SSd showed significant toxicity in high concentrations (Fig. 3B and D). Thus, the calcium influx induced by SSa and SSd in the calcium experiment could be false positive results. The values are the means of technical duplicates from three independent experiments with standard error.

### Compound specificity determination

**Figure 4:**
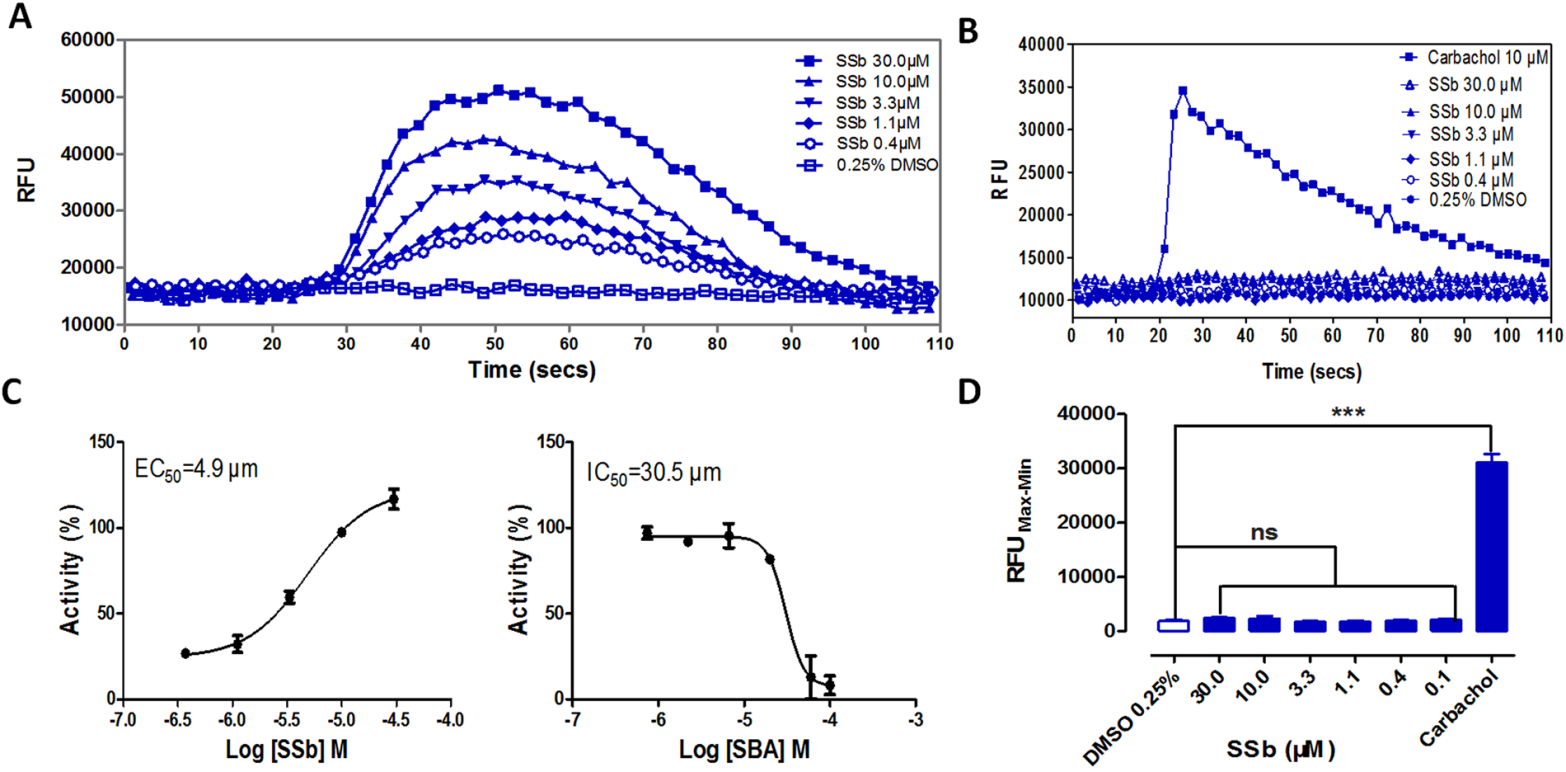
Hit verification in the HEK293-TAS2R14-G16gust44 cell line. (**A**) Calcium fluorescence signatures of HEK293-TAS2R14-G16gust44 cells treated with a concentration gradient of SSb over the course of 110 seconds (**B**) Agonist specificity of SSb. To observe whether SSb induced calcium influx in HEK293 host cells, the cells were treated with various concentrations of the tested compounds. Carbachol (10 μM) and 0.25% (v/v) DMSO were used as positive and negative controls, respectively. Calcium fluorescence signatures of HEK293 cells treated with a concentration gradient of SSb over the course of 110 seconds. (**C**) The EC_50_ value of SSb was calculated from concentration-response curves as 4.9 μM. The IC_50_ value of the antagonist SBA in the presence of 10.0 μM SSb was determined to be 30.5 μM. The maximum RFU value of TAS2R14 activation was set at 100%, and the minimum was set at 0%. (**D**) No statistically significant differences were observed between the SSb group and 0.25% DMSO group, suggesting that SSb specifically evoked a calcium signal as the second messenger signal downstream of TAS2R14. All error bars indicate the SE of three replicates. Bars with stars are significantly different from the control group, ***p < 0.05. RFU_Max-Min_ means the difference in relative fluorescence units between the maximum and the minimum. RFU: relative fluorescence units. The EC_50_ and IC_50_ values were determined in normalized RFU using GraphPad Prism 5 software.

To further evaluate the specificity of SSa, SSb and SSd, we conducted a calcium mobilization assay in TAS2R14 (−)-HEK293 cells. SSb did not show any effect on TAS2R14 (−)-HEK293 compared with a 0.25% DMSO group (Fig. 4B and D). However, SSa and SSd showed significant calcium trends in the TAS2R14 (−)-HEK293 cell line (data not shown). The results confirmed that the evoked calcium signal was the second messenger triggered by TAS2R14 activation upon exposure to SSb. SSb is a specific TAS2R14 agonist. The EC_50_ value of SSb in TAS2R14 agonist activity was determined to be 4.9 μM (Fig. 4A and C). The IC_50_ value of SBA, a known TAS2R14 antagonist, was determined to be 30.5 μM (Fig. 4C).

### IgE-induced mast cell degranulation

**Figure 5:**
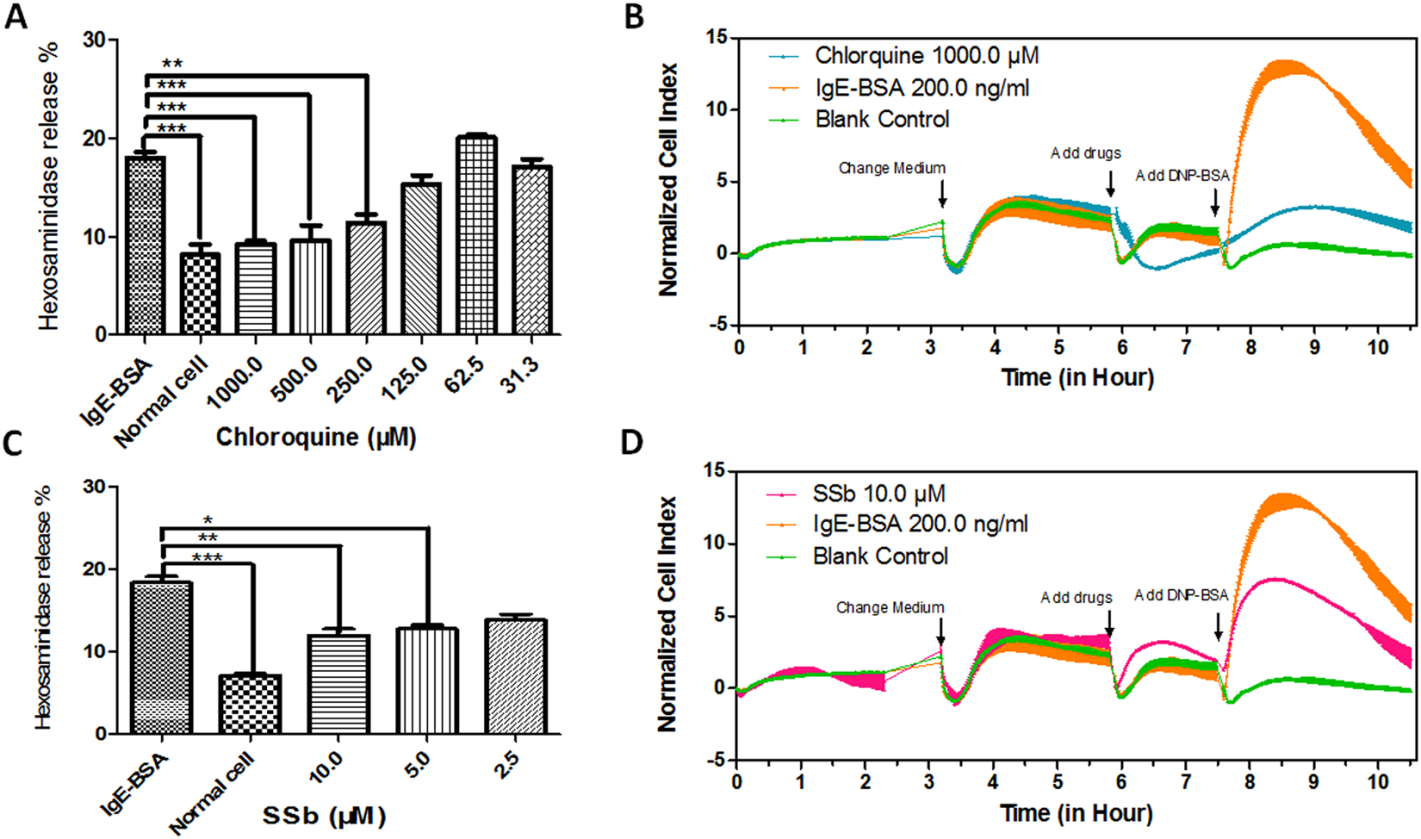
TAS2R14 agonists inhibited IgE-induced mast cell degranulation. (**A**) Chloroquine showed an inhibitory effect on IgE-dependent mast cell degranulation and served as a positive control in this study. Chloroquine significantly inhibited the IgE-induced release of β-hexosaminidase at 1000.0, 500.0, and 250.0 μM. (**B**) Meanwhile, the effect of IgE-induced mast cell degranulation was evaluated by cellular morphology. Chloroquine at 1000.0 μM could inhibit the IgE-induced increase in cell index as monitored by RTCA. (**C**) SSb showed inhibitory effect on the release of β-hexosaminidase at 10.0 μM and 5.0 μM. (**D**) SSb at 10.0 μM could inhibit the IgE-induced increase in cell index as monitored by RTCA. Bars with stars were significantly different from the control group, ***p < 0.05.

Beta-hexosaminidase is a granule marker for mast cell degranulation and was measured to evaluate the inhibitory effects of SSb on IgE-induced mast cell degranulation *in vitro*. As shown in Fig. 5A and C, chloroquine served as a positive control and significantly inhibit the IgE-induced release of β-hexosaminidase at 1000.0, 500.0 and 250.0 μM. In addition, SSb could inhibit the IgE-induced mast cell degranulation at 10.0 μM and 5.0 μM.

As an additional form of verification, the inhibitory effect of SSb on IgE-induced mast cell degranulation was also evaluated by cellular morphology using a real-time cell analysis (RTCA) assay. As shown in Fig. 5B and D, chloroquine (1000.0 μM) and SSb (10.0 μM) can significantly inhibit mast cell stimulation induced by dinitrophenyl-albumin conjugate (DNP-BSA) through monoclonal anti-dinitrophenyl antibody (anti-DNP-IgE). The degree of stimulation of mast cells is characterized as cell index (CI) monitored by RTCA.

### Binding site analysis between SSb and TAS2R14 model

**Figure 6:**
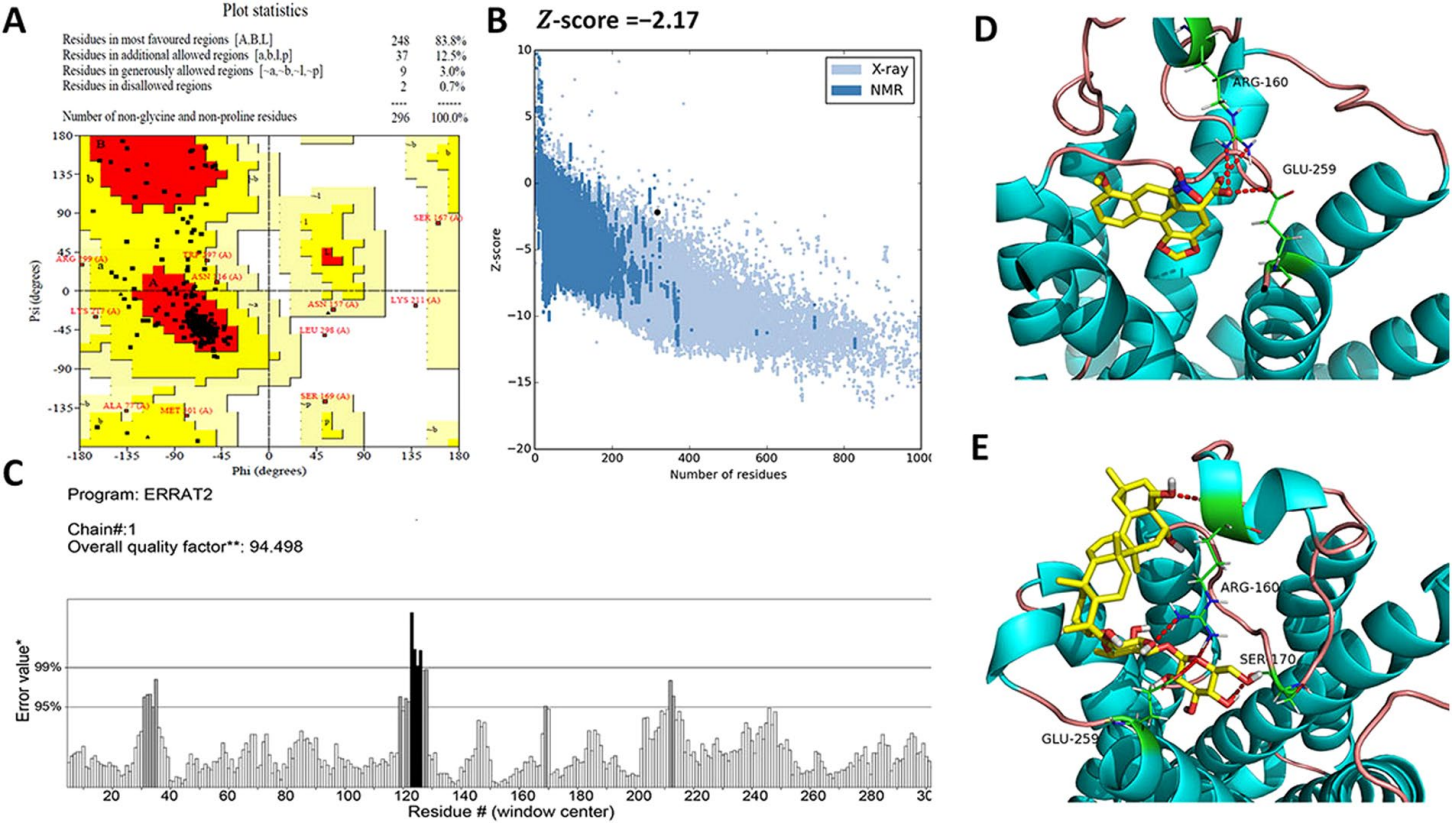
Three dimensional structural model establishment and molecular docking analysis. (**A**) Ramachandran plot of the constructed TAS2R14 model showed that 99.3% of the residues were in the allowed region; (**B**) *Z*-scores, processed by ProSA-web, were calculated according to the lengths of all protein chains in PDB as determined by X-ray crystallography (light blue) and NMR spectroscopy (dark blue), respectively. The *Z*-score in the light black dot for *TAS2R14_Model_1* was −2.17, which was in the range of native conformations of crystal structures; (**C**) The overall quality factor value of 94.5 (>90.00), checked by ERRAT, indicated a valuable model of TAS2R14. (**D**) Binding modes of TAS2R14 with AAA and SSb. The docking results implied that Arg160 and Glu259 of TAS2R14 were the key amino acid residues binding to AAA; (**E**) Arg160, Ser170 and Glu259 were the key amino acid residues binding to SSb. Regarding the interaction mode of SSb, the hydroxymethyl group at the C-28 position could specifically interact with TAS2R14 through hydrogen bonding. Hydrogen bonding interactions are displayed in dotted lines.

According to the five models generated, *TAS2R14_Model_1*, whose exp. TM-score and exp. RMSD were 0.71 ± 0.12 and 6.4 ± 3.9 Å, had the best C-score (−0.03) and was selected for molecular docking. A ramachandran plot showed that 83.8% of all residues are located in the most favored regions, 12.5% are in additionally allowed regions and 3.0% are in generously allowed regions (Fig. 6A). The average, root mean square (RMS) and distribution of Z-scores determined for TAS2R14 are shown in Fig. 6B. ERRAT produced an overall quality factor of 94.5 for TAS2R14 (Fig. 6C); the normally accepted range is above 90 for a high-quality model^26^. The results suggested that the established model of TAS2R14 established could be used for further studies.

Molecular docking results showed that AAA could bind to TAS2R14 via H-bond interaction with Arg160 and Glu259 (Fig. 6D). For SSb, Ser170 is another key amino acid residue involved in H-bond interactions (Fig. 6E). The total scores were calculated to be 2.60 and 4.50 for AAA and SSb, respectively (Table 3). The result is helpful for interpreting the mechanism of molecular recognition and for guiding structural optimization of ligands.

**Table 3:** Binding evaluation parameters of TAS2R14.

Names	Total Score	^a^Crash	^b^Polar
Aristolochic acid A (AAA)	2.600	−1.858	1.154
Saikosaponin B (SSb)	4.495	−5.498	3.338

^a^Crash represents the degree of unnecessary collisions caused by the ligand in the protein and self-clash between ligand atoms that are separated by rotatable bonds. The nearer Crash value to zero, the better the binding mode is. Negative values represent penetration; ^b^Polar represents the contribution of the polar interactions to the total score.

In terms of molecular structures, SSa, SSc and SSd are epoxy ether derivatives characterized by a 13,28β -epoxy bridge, while SSb is a heterocyclic diene derivative and has a hydroxymethyl group at the C-28 position. According to the simulation results of molecular docking analysis (Fig. 6E), the sugar chain of SSb could bind to TAS2R14 via three H-bond interactions with Arg160, Ser170 and Glu259. In addition, the hydroxyl group of SSb at the C-28 position could bind to TAS2R14 via H-bond interactions with Arg160, which is the only difference between SSb and other saikosaponins in this study.

## Discussion

Through *in vitro* evaluation, this study confirmed for the first time that SSb is a specific TAS2R14 agonist with no cytotoxicity, which implies that the bitterness of *Radix Bupleuri* may be an effect of activating TAS2R14. The combination of virtual screening, cell-based functional assays and binding studies performed in this work showed remarkable advantages in identifying direct TAS2R14 agonists from natural sources. The virtual screening provided a quick and economical identification of potential candidates from a large dataset of molecules. An intracellular calcium mobilization assay combined with cytotoxicity evaluation identified a specific and non-toxic agonist of TAS2R14. Moreover, the results implied that the potential mechanism of *Radix Bupleuri* in respiratory disease treatment might relate to its inhibition of mast cell degranulation.

Mast cells are important mediators of inflammatory responses such as allergy and anaphylaxis^27^. Degranulation is regarded as the main function of mast cells, with IgE as the main trigger^28^. SSb showed an inhibitory effect of mast cell degranulation, which could be proposed as a starting point for exploring whether it could show an anti-allergic asthma effect in animal models or clinic trials. But for all this, it still have limitations to determine the physiological activity of the SSb through the *in vitro* mast cell degranulation assay. An independent *in vitro* functional assay cannot completely reproduced the real environment of cells, but only investigate the activity of the SSb in inhibiting mast cell degranulation in a particular condition. Therefore, to determine the anti-asthma pharmacological activity of SSb, more *in vivo* experiments should be conducted to further verify its anti-asthmatic effect. In addition, the signal transduction mechanism deserves further study. In this study, SSb was confirmed as a direct TAS2R14 agonist, but whether SSb can activate the other 24 hTAS2Rs is still unknown.

Compared with prototypic bitter compounds for the 20 TAS2Rs deorphaned in a previous study^29^, SSb is the first known TAS2R14 agonist with its particular chemical skeleton. In this work, ligand-based virtual screening was used for identification of TAS2R14 agonists from *Radix Bupleuri*. Three subtypes of saikosaponin (SSa, SSb and SSd) were identified as hits by the pharmacophore model. These compounds have a common parent structure but have some minor differences. SSa and SSd are epoxy ether derivatives, whose 13,28β-epoxy ether bond is their key characteristic. SSb is a heterocyclic diene derivative and has a hydroxymethyl group at the C-28 position. Because of the minor structural variation, SSb was confirmed through *in vitro* evaluation to be a specific TAS2R14 agonist with no cytotoxicity. This result is consistent with the widespread belief that structure decides the properties and function of biomolecules^30,31^. Such specific structural characteristics of SSb provide a valuable reference for further structural modification in drug design. TAS2R14 is an example of a G protein-coupled receptor; proteins that are generally considered to have complex intracellular signaling pathways. Different agonists are able to activate the same receptor, to elicit different receptor conformation, to direct different signaling responses. The reason may be that the structural diversity of different agonists leads to different binding modes and different active conformation of the target protein. Thus, binding analysis for the newfound agonist is important in further research. This study revealed the key binding characteristics of SSb in activating TAS2R14. Moreover, plants are natural resources of medicines. There are many chemical components in *Radix Bupleuri*, and most of them are present at low concentrations. Pharmacophore-based virtual screening helps to narrow down the field of potential agonists, which not only drastically reduces isolation and purchasing costs, but also greatly improves the efficiency of testing.

Human bitter taste receptors have attracted much attention over the past several years. Indeed, relevant research has continued ceaselessly because of the major importance of feeding and tasting, which is one of the essential elements of human nature. Bitterness is more unacceptable than the other tastes. Recently, an increasing amount of research has claimed that TAS2Rs show a physiological function or curative effects. As investigation continues into the involvement of TAS2Rs in diseases and their use as therapeutic targets, identifying potent and specific agonists will become increasingly valuable as a new solution to treat diseases.

## Materials and Methods

### Chemical and reagents

All the test compounds were acquired from the National Institutes for Food and Drug Control (Beijing, China) with purities greater than 98%. SBA, a known antagonist of TAS2R14, was prepared by Prof. Ouyang in The Third Military Medical University. AAA, trypsin, carbachol, hygromycin B, probenecid and acid red 1 (purity ≥98%) were all obtained from Sigma-Aldrich (St. Louis, MO, USA). Anti-DNP IgE and DNP-BSA were purchased from Sigma Aldrich (St. Louis, MO, USA). Fluo-4 AM was purchased from Molecular Probes (Grand Island, NY, USA). Matrigel was purchased from Becton Dickinson (New York, NY, USA). Dulbecco’s modified Eagle’s medium (DMEM) and fetal bovine serum (FBS) were obtained from Gibco BRL (Grand Island, NY, USA). A CellTiter-Glo Luminescent Cell Viability Assay kit was purchased from Promega (Madison, WI, USA). 3D chemical database of *Radix Bupleuri* a total of 155 compounds isolated from *Radix Bupleuri* were collected from Traditional Chinese Medicine Database 2009^32^ (TCMD 2009). The structures of all the molecules were extracted and saved in the Mol2 format. All the structures were converted to 3D conformers through the CONCORD module in SYBYL X -1.2 software (Tripos Inc., St. Louis, MO, USA). Afterwards, the structures were checked and energy optimized using the Tripos force field (Powell method and 0.05 kcal/mol Å energy gradient convergence criteria). Then, they were stored as RBDB.

### Pharmacophore model-based virtual screening

Twelve compounds^33–36^ were used as a training set to generate the pharmacophore models of TAS2R14 agonists using a *common feature pharmacophore generation* protocol in Discovery Studio v3.5 (Accelrys, San Diego, CA, USA), in considering the agonistic activity and structural diversity of the known TAS2R14 agonists. Another 37 experimentally known TAS2R14 agonists^34^ were used to validate the models. Pharmacophore models of TAS2R14 agonists were generated according to our previous studies^22^.

An external database with a decoy set, consisting of 37 experimentally known TAS2R14 agonists^34^ and 191 non-active compounds^37^, was used to validate the pharmacophore models established. Four parameters (A%, Y%, N and CAI) proposed in our previous work^23^ were applied to assess the performance of the pharmacophore models. Ht represents the number of hits, and Ha represents the number of active hits. The letter D represents the number of compounds in the external database, and A represents the number of active compounds. A% represents the ability to identify active compounds from the external database. Y% represents the proportion of active hits in total hits. N (namely, the identified effective index) represents the ability to distinguish active compounds from non-active compounds. CAI, comprehensive appraisal index, was proposed to evaluate the models comprehensively. The model with the highest CAI was selected as a 3D query to screen RBDB using the Ligand Profiler protocol in Discovery Studio v3.5. The *minimum interference distance* was set at 1 Å, and the *search method* was set at *Best*. The other protocol parameters were left at the default settings. The fit value was calculated to indicate the matching degree between each ligand and the pharmacophore features. A higher fit value suggests a better alignment between the ligand’s conformer and the pharmacophore model.

### HEK293-TAS2R14-G16gust44 cell line establishment

To determine the TAS2R14-agonistic activity of the hits from virtual screening, we established an HEK293-TAS2R14-G16gust44 cell line. Gα16 protein fused with 44 amino acids of gustducin is indispensable for the detection of Gαi-coupled TAS2R activity in calcium assays^38^. The full-length human TAS2R14 cDNA was cloned and then co-transfected along with Gα16 protein fused with 44 amino acids of gustducin into the HEK293 cells. A stable recombinant HEK293-TAS2R14-G16gust44 cell line with a high signal/background ratio under stimulation with AAA was obtained for further assay development and validation. Moreover, the robustness of this assay was evaluated using the Z′ factor^25^. Value 0.5 ≦ Z′ < 1.0 are recommended as an indication of proper assay optimization^39^. AAA (10 μM) and 0.25% (v/v) DMSO served as positive and negative controls, respectively. The Z′ factor was defined as follows:

1 $${\rm{Z}}^{\prime} ={\rm{1}}-\frac{3\times {\rm{SD}}\,{\rm{of}}\,{\rm{positive}}\,{\rm{control}}+3\times {\rm{SD}}\,{\rm{of}}\,{\rm{negative}}\,{\rm{control}}}{{\rm{mean}}\,{\rm{of}}\,{\rm{positive}}\,{\rm{control}}-{\rm{mean}}\,{\rm{of}}\,{\rm{negative}}\,{\rm{control}}}$$

### Intracellular calcium mobilization assay

The HEK293 cells were maintained in DMEM containing 10% FBS, 100 U/mL penicillin, and 100 μg/mL streptomycin in a humidified atmosphere of 5% CO_2_ at 37 °C. The HEK293-TAS2R14-G16gust44 cells were then incubated with complete culture medium along with 50 μg/mL of hygromycin B and 400 μg/mL of G418. An HEK293-TAS2R14-G16gust44 cell-based intracellular calcium mobilization assay was designed to evaluate the TAS2R14-agonistic activity of the hits. The HEK293-TAS2R14-G16gust44 cells were seeded at a density of 3.0 × 10^4^ per well into 96-well clear-bottom black plates coated with Matrigel and were incubated in 5% CO_2_ at 37 °C overnight. On the day of the assay, the growth medium in each well was replaced with 100 μL of loading buffer containing a final concentration of 4 μM Ca^2+^-sensitive Fluo-4 AM dye, 2 mM acid red 1 and 2.5 mM probenecid in HBSS. The plate was then incubated at 37 °C in the dark for 30 min before the calcium signal was read out. For the antagonism study, 80 μL of loading buffer was added to each well along with 20 μL of HBSS containing the appropriate test compound was added 10 min prior to calcium-flux measurement. Cells were transferred to a FlexStation II (Molecular Devices) for experimentation. Basal fluorescence was recorded for 16 s before the agonist was applied. The integrated FlexStation II fluidics system transferred 25 μL of the compound (5 × solution) from the agonist compound plate to the assay plate, which contained 100 μL of loading buffer solution. The FlexStation II read relative fluorescence units (RFU) at 37 °C with an excitation wavelength of 485 nm and an emission wavelength of 525 nm. The fluorescence intensity was read every 2.00 s for 100 s.

In this study, 60 μM AAA and 0.25% DMSO were used as the positive and negative control respectively. All the test compounds were dissolved in DMSO at 36 mM. The final DMSO concentration in each well was controlled at 0.25% for all the tested compounds. The EC_50_ and IC_50_ values were determined in normalized RFU by GraphPad Prism software (version 5, GraphPad Software, Inc., USA). Each data point represents the mean ± SEM. The data were analyzed using one-way analysis of variance (ANOVA) followed by Dunnett’s multiple comparison tests to analyze the differences between group means; p < 0.05 was considered significant.

### Cytotoxicity assay

HEK293-TAS2R14-G16gust44 cells were seeded at 3.0 × 10^4^ per well in 96-well clear-bottom black plates and incubated in 5% CO_2_ at 37 °C overnight. Different concentrations of the natural chemicals and a solution of 0.25% DMSO was added to the 96-well plates and incubated in 5% CO_2_ at 37 °C for 24 hours. A concentration of 0.02% Triton was added 10 min before detection for experimental sensitivity evaluation. Luminescence was read by Envision 2100 after incubation with CellTiter-Glo reagent for 15 min.

### Specificity determination

A specificity assay was conducted to eliminate the false positive compounds from the functional evaluation. If a compound has the same ability to induce transient calcium influx in HEK293 host cells and HEK293-TAS2R14-G16gust44 cells, this compound should be considered a false positive. The cells were treated with various concentrations of tested compounds to observe whether the identified compounds induced calcium influx in HEK293 host cells. Carbachol, a P2Y receptor agonist that can elicit intracellular calcium mobilization^40^, was used as a positive control in HEK293. Meanwhile, the 0.25% DMSO solution was used as a negative control.

### IgE-induced mast cell degranulation assay

RBL-2H3 cells were maintained in EMEM containing 10% FBS, 100 U/mL penicillin, and 100 μg/mL streptomycin in a humidified atmosphere of 5% CO_2_ at 37 °C. RBL-2H3 were grown in 96-well plates (2 × 10^4^ cells/180 μL/well) for one day before being sensitized overnight with 20 μL of anti-DNP-IgE (final concentration: 200 ng/ml). The cells were then washed twice with PBS buffer. We added different concentration of compounds dissolved in DMEM (no phenol red) and incubated the cultures for 1 hour at 37 °C. After incubation, the cells were stimulated at 37 °C with 200 ng/mL DNP-BSA, incubated for 30 min, and then cooled to 0 °C in an external ice bath for 10 min. Then, the culture supernatant (50 μL) was incubated for 1.5 hours at 37 °C with 100 μL of p-nitrophenyl-N-acetyl-β-D-glucosaminide (1 mM/L, Sigma, N9376) in 0.1 mol/L sodium citrate buffer (pH 4.5). Afterwards, the reaction was terminated by the addition of 200 μL of 0.1 mol/L sodium carbonate buffer (pH 10.5). The generation of p-nitrophenol was monitored by measurement the absorbance at 405 nm. To determine the total amount of β-hexosaminidase released, the remaining cells were lysed by treatment with assay buffer containing 0.1% (v/v) Triton X-100 prior to incubation with the substrate using the same procedure as for the determination of activity in the supernatant. The percentage of β-hexosaminidase released (%) was calculated as follows: % = (A_up_ − A_0_)/(A_whole_ − A_0_) × 100. A_0_ is the absorbance of the un-stimulated cells in the supernatant, A_up_ is the absorbance of the stimulated cells in the supernatant and A_whole_ is the absorbance of the total cell lysate.

Real-time assays were performed with an xCELLigence RTCA SP instrument according to the instructions of the supplier (ACEA Biosciences)^41,42^. An E-Plate 96 was coated with 50 μL of a 1:100 diluted Matrigel solution and incubated overnight at 4 °C. RBL-2H3 cells were grown in 96-well plates (2 × 10^4^ cells/180 μL/well) for one day before being sensitized overnight with 20 μL of anti-DNP-IgE (final concentration: 200 ng/ml). The E-plate was monitored every 15 min on the RTCA Cardio Instrument at 37 °C in a 5% CO_2_ incubator. The cells were then washed twice with PBS buffer after 24 hours of incubation with anti-DNP-IgE incubation. We added different concentration of compounds dissolved in EMEM (no FBS) and incubated the cultures for about 1 hour at 37 °C until the cell index had plateaued. After incubation, the cells were stimulated at 37 °C with 200 ng/mL DNP-BSA. After treatment, samples were measured every minute for 1 hour. Chloroquine, a known TAS2R agonist, that inhibits IgE-dependent mast cells degranulation^43,44^, was used as a positive control.

### Binding mode of TAS2R14 with SSb

As the crystal structure of TAS2R14 has not yet been resolved, a 3D structural model of TAS2R14 was constructed through the I-TASSER server; the SPICKER program computed possible models corresponding to the five largest structure clusters^45,46^. The human TAS2R14 amino acid sequence (UniProtKB-Q9NYV8) was obtained from the National Center for Biotechnology Information (NCBI, http://www.ncbi.nlm.nih.gov) in FASTA format^47^. The amino acid sequence of TAS2R14 was submitted to the I-TASSER server to determine 3D structure^45,46^. The output of I-TASSER was analyzed using PyMol program^48^. Confidence score (C-score) and template modelling score (TM-score) were used as the parameters to rank the quality of the modeled structures. A C-score value typically ranges from −0.2 to 5, and a higher C-score indicates a model with higher confidence. A model with a TM score >0.5 is considered to show significant topology with the template homolog. The TAS2R14 model with the highest C-score and TM-score was selected for structural analysis. The model was further assessed using PROCHECK^49^ and ERRAT^50^ through the SAVES server (http://services.mbi.ucla.edu/SAVES/). The overall stereochemistry of each residue of *TAS2R14_Model_1* was analyzed using ramachandran plot. ProSA-web was used to check the 3D model of *TAS2R14_Model_1* for potential errors^51^. The *Z*-score indicates the overall quality of the model and measures the deviation of the total energy of the structure with respect to an energy distribution derived from random conformations.

The Multi-Channel Surfaces search protocol in SYBYL X-1.2 was used to search the cavities on the surface of the protein. Considering the binding site mutagenesis experiments reported from the previous literature^52^, the cavity containing active amino acid residues was selected as the active pocket surface to generate a ProtoMol model for molecular docking. The ProtoMol model was generated using the steric hydrophobic group (CH_4_), the hydrogen bond group (C=O), and the hydrogen acceptor (N-H) within 4.5 Å of the active pocket surface.

Surflex-Dock, one of the well-recognized methods in the molecular docking field^53–55^, was employed to perform virtual screening and study the ligand-receptor interaction. To check the accuracy of the docking program, AAA was docked into the active site of TAS2R14. After validation, SSb was prepared according to the following procedure: (1) The structural correctness was checked, hydrogen atoms and atomic charges were added by the Gasteiger-Hückel method, energy minimization was performed using the Tripos force field for 1000 iterations. (2) Each optimized compound was docked into the active site of TAS2R14 using the default settings, respectively. (3) After each Surflex-Dock run, the best ten docked conformers or poses were sorted in a molecular spreadsheet, and they represented binding affinities in log 10(Kd) based on the Surflex-dock scoring function (crash score (also pKd units), polar score, D-score, PMF-score, G-score, ChemSco and CScore)^56^.

## Conclusion

*Radix Bupleuri* is a common Chinese herbal medicine for respiratory disease. To explore the bitter ingredients and their relationships with the clinical effect of *Radix Bupleuri*, this study screened natural TAS2R14-targeted bitter tastants and evaluated the inhibitory function on mast cell degranulation. SSb was ultimately confirmed as a specific agonist of TAS2R14. A cytotoxicity bioassay indicated that SSb showed no significant cytotoxicity to HEK293 cells within 24 hours. Molecular docking showed that SSb could directly bind to the TAS2R14 model through H-bond interactions with Arg160, Ser170 and Glu259. Moreover, SSb showed the ability to inhibit IgE-induced mast cell degranulation according to a β-hexosaminidase release model and real-time cell analysis. This study demonstrated that SSb is a direct TAS2R14 agonist that inhibits IgE-induced mast cell degranulation. However, owing to the restrictions of *in vitro* functional assay, more *in vivo* studies should be conducted to further verify the anti-asthma activity of SSb.
